# A randomised controlled implementation trial of the feasibility and effectiveness of school staff delivery of a selective substance use and mental health program during the COVID-19 pandemic

**DOI:** 10.1186/s12889-025-21493-1

**Published:** 2025-01-28

**Authors:** Lucinda Grummitt, Kirsty Rowlinson, Joanne Cassar, Chloe Conroy, Louise Birrell, Lexine Stapinski, Emma Louise Barrett, Julia Macauley, Maree Teesson, Nicola C. Newton, Erin V. Kelly

**Affiliations:** The Matilda Centre for Research in Mental Health and Substance Use, The University of Sydney, Level 6, Jane Foss Russell Building, Sydney, NSW 2006 Australia

**Keywords:** Prevention, Alcohol use, Mental health, Personality, School, Randomised controlled trial, Selective, Implementation

## Abstract

**Background:**

*Preventure* is a selective school-based personality-targeted program that has shown long-term benefits in preventing student alcohol use, internalising and externalising problems when delivered by psychologists. In this first Australian randomised controlled trial of school staff implementation of *Preventure*, we aimed to examine i) acceptability, feasibility, and fidelity and ii) effectiveness of *Preventure* on student alcohol use, internalising, and externalising symptoms.

**Methods:**

A cluster-randomised controlled implementation trial was conducted in Sydney, Australia and was guided by the RE-AIM framework (Glasgow et al. 1999); which measures reach, effectiveness, adoption, implementation, and maintenance. Schools were randomly assigned to either the Preventure intervention or active control (health education as usual). Nominated school staff from intervention schools received training and delivered the program to students with elevated scores on one of four personality types targeted in the program. School staff completed surveys on RE-AIM measures, which were analysed using descriptive statistics and thematic analysis. Students completed surveys at baseline and six months post-intervention; mixed-effects regression examined intervention by time interactions on alcohol use, internalising and externalising problems, at six-month follow-up. The study was prospectively registered with the Australian New Zealand Clinical Trials Registry (ACTRN12620000790943, registration date 6 August 2020).

**Results:**

553 students across 8 schools participated in the baseline survey. Of these, 40% had elevated scores on one of the four personality profiles, resulting in 220 students in the current study (102 students in intervention schools, 118 students in control schools; mean age 13.6, 45.7% female). Most RE-AIM domains showed high ratings, with school staff showing good adherence and confidence in delivery. However, teachers reported difficulties with feasibility, particularly a lack of time. Student outcomes: There were significant improvements in depression and conduct problems across both intervention and control across time. There were no significant main or interaction effects of the intervention on student alcohol use, internalising, or externalising problems.

**Discussion:**

Teachers and students rated the program highly. However, concerns around feasibility may limit teacher-led application of the program in the Australian context. Alternative approaches, such as delivery by dedicated personnel within schools without a teaching load, may be critical in implementing such evidence-based interventions at scale.

**Trial registration:**

The study was prospectively registered with the Australian New Zealand Clinical Trials Registry, registration number: ACTRN12620000790943, registration date: 6 August 2020.

**Supplementary Information:**

The online version contains supplementary material available at 10.1186/s12889-025-21493-1.

## Introduction

Globally, almost half (48%) of all mental and substance use disorders have their onset before the age of 18, and these disorders are the leading cause of disease burden among young people [10, 37]. Schools have been identified as optimal settings for the delivery of interventions to prevent the onset and escalation of substance use and mental disorders, given broad reach to many adolescents, mandated health education, and established referral pathways for students requiring greater support [6, 13]. However, it is also well-recognised that school staff are severely overburdened, with heavy workloads and an ever-expanding list of topics seen as within their remit [26]. This became particularly salient during the COVID-19 pandemic, where teachers faced significant challenges and were described as “the forgotten frontline workers” [3]. In the wake of the pandemic, understanding how to best address student mental health in schools without further burdening school staff is critical.

Selective prevention, delivered to those identified at higher risk, has shown promising reductions in mental health and substance use outcomes among students [8, 40, 43]. This approach enables the intervention to be tailored to addressing risk factors that may be relevant for a subsample of the population and thus provide greater intensity content and skills training directed to these individuals. Accordingly, selective prevention programs show consistent efficacy in symptom reduction and delayed onset, with effects sustained long-term (i.e., up to 7 years post-intervention) [31]. However, selective prevention comes with implementation challenges that need to be managed in school settings, including the potential for stigma or labelling from identifying some students as at greater risk of mental health problems, as well as practical issues to do with scheduling intervention sessions for some students within a cohort.

Further, school-based prevention programs have typically targeted single disorders, such as depression, anxiety, or alcohol misuse, despite evidence of substantial comorbidity between disorders and overlapping aetiology [16, 24]. In contrast, transdiagnostic approaches to the prevention of substance use and mental disorders hold great promise. Such approaches are built on the knowledge that common mental disorders, such as internalising (e.g., depression, anxiety), externalising (e.g., conduct disorder, hyperactivity), and substance use disorders, share common risk factors [22, 27], and that addressing shared risk factors can more effectively prevent these disorders, compared to addressing them in isolation [23, 36, 39]. Transdiagnostic approaches may be immensely beneficial for schools as an efficient way to address multiple student concerns within time and scheduling restraints.

One transdiagnostic, selective prevention program for substance use and mental disorders is the *Preventure* program [8, 9]. *Preventure* targets four personality traits (two internalising, two externalising) that have been identified as risk factors for problematic alcohol use: impulsivity, sensation seeking, anxiety sensitivity, and hopelessness/negative thinking [7]. It is a brief, school-based intervention that is delivered by clinical psychologists to students high in one of these traits. *Preventure* has been evaluated extensively through randomised controlled trials in Australia and internationally, which have demonstrated reductions in alcohol use, binge drinking, alcohol-related harms, symptoms of depression, anxiety, hyperactivity, conduct problems, and suicidal ideation for up to three years post-intervention, compared to active control (health education as usual)[8, 14, 30, 32, 40]. Moreover, reductions in problematic alcohol use have been shown for up to seven years post-intervention, compared to active control [31].

In the aforementioned trials, the intervention was delivered by psychologists external to the school. However, outside a research environment, many schools do not have the resources to contract psychologists to deliver a prevention program to their students. Barriers such as cost, the availability of psychologists in the school’s area, and access to training, result in inequitable access to evidence-based prevention. Upskilling staff already embedded in the school could overcome many of the barriers to program access, including cost and geographic restrictions, enabling this efficacious program to be implemented equitably and at scale. To date there has only been one reported trial in the UK of teacher delivered *Preventure.* This trial produced similar reductions in depression, anxiety, and conduct problems, as psychologist-delivered *Preventure,* however, substance use outcomes were not measured [33]. Moreover, whether teachers found the intervention acceptable and feasible to deliver was not measured. In addition, since 2010 when this trial concluded, both student mental health concerns as well as demands on teachers have steadily increased [26, 20], raising the question of whether teacher delivery of Preventure would be feasible and acceptable and produce benefits for students as demonstrated in the UK trial of Preventure in a contemporary context [33].

The feasibility and acceptability of delivering interventions in real-world practice is critical area of health research. Even the most effective interventions have limited value if they cannot be successfully delivered in practice and sustained in the long-term. Implementation success has been frequently measured by the RE-AIM framework [11], which spans 5 key dimensions: reach, effectiveness, adoption, implementation, and maintenance. Reach refers to the number, proportion, and diversity of individuals who are willing to participate in the intervention. Effectiveness measures the intervention's impact on outcomes, including potential adverse effects, quality of life, and economic results. Adoption considers the number, proportion, and diversity of settings and facilitators that implement the program, and implementation reflects how well facilitators adhere to the protocol. Finally, maintenance considers the extent to which a program becomes part of routine practice.

The current study was an implementation randomised controlled trial (RCT) guided by the RE-AIM framework to examine effectiveness, feasibility, acceptability, and fidelity of the Preventure program when delivered by school staff. Primary outcomes were school staff-rated feasibility and acceptability of delivery the intervention, assessed with the RE-AIM framework. Pre-registered effectiveness hypotheses were that compared to students with elevated personality scores in control schools, students with elevated personality scores in *Preventure* schools would exhibit significantly decreased i) alcohol use, intentions to use alcohol, alcohol-related harm, ii) anxiety and depressive symptoms, and iii) conduct problems and hyperactivity symptoms at six and 12-months post-baseline [19]. The trial initially planned to begin recruitment of schools and students in March 2020, which coincided with the declaration of the COVID-19 pandemic in Australia. The trial proceeded as planned but significant flexibility was built into trial decision making to allow for the extra challenges faced by schools and students at this time. Therefore, findings should be understood within the broader context of the unfolding pandemic. This was undoubtedly a sub-optimal time to test the question of whether school staff can effectively implement Preventure, however, this unique context provides insights into the impact of the pandemic on research and implementation of school-based mental health and substance use interventions.

## Methods

The School-led Preventure trial was a CONSORT-compliant, cluster RCT conducted in secondary schools in greater Sydney, Australia, from 2020—2022. The trial protocol was pre-registered with the Australian and New Zealand Clinical Trials Registry (ACTRN12620000790943, registration date: 6 August 2020). In accordance with the Declaration of Helsinki and Australian National Statement on Ethical Conduct in Human Research, ethical approval was obtained from the University of Sydney Human Research Ethics Committee (2019/792), and the approval body for New South Wales (NSW) public (state) schools (State Education Research Applications Process). Full details of the original study design, recruitment methodology, and informed consent are published in the study protocol [19].

### Sample and procedures

Schools in the greater Sydney region, NSW, Australia, were invited to participate in the trial via email and phone using publicly available contact details. Recruitment was delayed by three months and extended due to COVID-19 impacts on schools (such as remote learning and logistical challenges to schools during this time). Following school principal agreement to participate, a researcher external to the study block-randomised schools to either *Preventure* or active control (health education as usual) using the Blockrand package in R (Snow, 2020) stratified by majority gender (ie > 60% male or female). Principals of schools who were randomised to the intervention group identified two to four staff members interested in delivering the Preventure program (henceforth, facilitators and co-facilitators) and coordinating the school’s participation in trial activities. Voluntary and informed written consent was obtained from students and their parents, and the staff members delivering the Preventure program.

As per the pre-registered protocol, intention-to-treat analyses were planned for all primary outcomes. The trial was powered to detect an effect size of 0.3 (*p* = 0.05) in a sample of 280 students with elevated personality scores at the final timepoint. Due to the COVID-19 pandemic and resulting school withdrawals, this sample size was not achieved, with 220 students with elevated personality scores at baseline, and 48% attrition across the study, resulting in 115 students retained at the final timepoint.

The original study design included three assessment periods for Year 8 and Year 9 students in both intervention and control groups: an online self-report survey at baseline (before the intervention), post-test (roughly 6 months post-baseline), and 12 months post-baseline. However, the timelines for the baseline and six-month follow-up were substantially delayed by COVID-19 impacts, as shown in Table 1. These delays necessitated a deviation to the study protocol, cancelling the 12-month follow up survey. Follow-up periods between the baseline and post-test survey ranged from 6 to 16 months; while every effort was made to adhere to study timelines, greater Sydney went through a series of stay-at-home orders during the study period which severely disrupted schools’ ability to complete follow-up assessments according to a pre-planned schedule.

**Table 1:** Baseline characteristics of the intention to treat sample, *N* = 220

Characteristic	Control (*n* = 118)	Preventure (*n* = 102)
No. of schools	5	3
Age, mean (SD)	13.8 (0.6)	13.4 (0.7)
Age, median	14	13
Sex, n (%)		
Male	60 (50.8)	47 (45.6)
Female	49 (41.5)	52 (50.5)
Non-binary	7 (5.9)	3 (2.9)
Other	2 (1.7)	0 (0.0)
Personality profile, n (%)		
Negative thinking	44 (37.3)	31 (30.1)
Anxiety sensitivity	27 (22.9)	38 (36.9)
Impulsivity	14 (11.9)	16 (15.5)
Sensation seeking	33 (28.0)	18 (17.5)
Family affluence		
Mean (SD)	6.1 (1.6)	6.5 (1.4)
Low, n (%)	4 (3.4)	0 (0.0)
Medium, n (%)	32 (27.1)	22 (21.6)
High, n (%)	82 (69.5)	80 (78.4)

Expressed as No. (%) unless otherwise indicated

To maintain confidentiality, students entered a unique identifier at each survey assessment, which was used to link their responses over time. To ensure maximum retention, school staff contacted any absent students to offer an alternative time to complete the survey. Moreover, students who completed the surveys were entered into a prize draw to win a $50 gift voucher for each survey they completed. Staff involved in the study in both intervention and control schools received a reimbursement of $50 for their time at the end of the study.

### Facilitator training

School staff were trained as facilitators and co-facilitators according to the training protocol described in O’Leary-Barrett et al. [33]. This included a 2-day training workshop, followed by supervision to ensure adherence to the intervention. Due to COVID-19 restrictions, training was a hybrid of online and in-person (one day for each). All facilitators were provided with facilitator manuals to assist in their delivery of the program. School staff from the control schools were offered free Preventure training and program materials once they had completed the follow-up assessment.

### Intervention

For schools assigned to the intervention condition, students were screened using the Substance Use Risk Profile Scale (SURPS; Woicik et al., 2009), as per standard Preventure protocol [8]. Specifically, students with scores greater than one standard deviation above the population mean on any of the SURPS subscales (anxiety sensitivity (AS), negative thinking (NT), sensation seeking (SS), and impulsivity (IMP)) at baseline were invited to participate in Preventure and were allocated to the group for the corresponding personality trait (i.e. either the AS, NT, SS or IMP group). Each student was only able to participate in one personality-matched group; students with high scores on more than one SURPS subscale were allocated to the group in which they deviated the most from the population mean.

The Preventure intervention involves two 90-min sessions per personality trait, run in small groups of students led by trained facilitators. In each personality-targeted group, students learn about the target personality trait (e.g. common ways of thinking, feeling and acting related to that trait, including risky ways of coping), set goals, and learn skills to help them to cope with unhelpful aspects of the personality trait and to assist them in achieving their goals. The program was delivered in addition to the standard Health and Physical Education curriculum for Year 8/9 students, which includes content on substance use and mental wellbeing. Control schools implemented the usual Health and Physical Education curriculum for Year 8 and/or Year 9, which included content on mental health and wellbeing, as well as alcohol and other drugs. After the study, control schools were provided with the opportunity to undertake training in Preventure and deliver the program in their school.

### Measures

#### RE-AIM outcomes

At the end of the *Preventure* program, intervention staff completed an evaluation survey that assessed perspectives on program feasibility and acceptability. Staff evaluation surveys used a combination of open- and closed-ended questions based on the RE-AIM model [11] to assess staff perspectives on effectiveness, adoption, implementation, and maintenance of the Preventure program. The full survey can be found in the supplement.

### Reach

Reach was determined as the overall participation rate in the *Preventure* program. That is, of those students in *Preventure* schools who were invited to participate, how many completed the intervention.

### Perceived Effectiveness

Effectiveness was measured through i) staff and ii) student perspectives.

i) Staff perspectives: Staff were asked a series of questions on their perceived effectiveness of the program. Specifically, they were asked to rate on a 5-point Likert-type scale, “*To what extent do you believe the students benefitted from the Preventure program?; How effective do you think the Preventure program was for providing coping skills?; Do you think students will use the skills they learnt?; How would you rate the overall level of engagement among students?; Overall, how useful do you feel the Preventure program is?;* and* Did you notice any negative effects that could be attributed to the program?”*
ii) Student perspectives

The student feedback survey included open and closed questions assessing what students liked or disliked about the program, how helpful they found the program, and the relevance of content.

### Teacher-perceived Adoption

Adoption measured the number and representativeness of the facilitators and intervention settings in which Preventure could be delivered. This was assessed through a series of items assessing the staff members perspective (e.g., teacher, school counsellor, other), years of experience working in schools, do they work with young people in any other settings – and if so, would it be feasible to deliver *Preventure* in these settings.

### Implementation

Adherence to the intervention was collected via i) a self-report form completed by the main facilitator, ii) a report made by the co-facilitator, and iii) self-report questions to gage their overall perspectives on how easy or difficult it was to adhere to the intervention.

i) Self-report fidelity by the main facilitator was captured via 5 Likert-type items (from 1-Strongly Disagree to 5-Strongly Agree): *Established a good rapport with the students; Used language and vocabulary that was easily understood by students; Allowed students to express their views on situations freely and without immediately challenging them; Used empathic statements and a non-judgmental approach toward students; Used indirect or "conversational" questioning techniques where appropriate.* A total score was created with a maximum total of 25, with higher scores reflecting greater fidelity.
ii) Co-facilitators were asked to rate the facilitator on four quantitative items for each of the 2 Preventure sessions, assessing which parts of the intervention were covered (12 items for session 1; 7 items for session 2), overall facilitator adherence (from 1-Not at all to 5-Total adherence), overall level of student concentration (1-Very Poor to 5-Very Good) and engagement (1-Completely Disengaged to 4-Completely Engaged). Three qualitative items were asked as to whether the facilitator made any changes to the intervention, the reasons for this, and any external interference or barriers to adherence they encountered. The full measure can be found in the supplement. The quantitative items were summed to create a total “Co-facilitator-rated adherence” score of 26 for session 1 and 21 for session 2.
iii) Self-report questions consisted of the following: “*How confident do you feel in your ability to implement the Preventure program?; How difficult or easy did you find adhering to the manual?; Was there anything that made it challenging to adhere to?; Do you think the Preventure program could be implemented in a range of settings?”*

### Teacher anticipated Maintenance

Maintenance was assessed through the following items relating to ongoing delivery of the program within the school:*“Will the Preventure program be implemented in your school in an ongoing basis?; Will you continue to be involved in the delivery of Preventure?; Do you think the Preventure training workshop was sufficient to enable you to continue delivering Preventure without further training?; Are there any barriers to the continued implementation of Preventure in your school?; To what extent do you believe Preventure will become an established program in your school?”.*

#### Student-level effectiveness outcomes

### Alcohol use and related harms

A series of questionnaires from the School Health and Alcohol Harm Reduction Project 'Patterns of Alcohol' index [25] were used to measure three alcohol outcomes. Students’ intentions to try alcohol were measured with a single item asking them to rate how likely they think it is that they will try alcohol at any time in the future (from Very Unlikely to Very Likely, or they have already tried alcohol). Students were asked whether they had consumed a standard drink in the past 6 months and were provided with a chart demonstrating the number of standard drinks contained in various alcoholic beverages e.g. can of ready-to-drink spirits, glass of white wine. Finally, students were asked how often they had consumed five or more standard drinks on one occasion over the past six months (from Never to Daily or Almost Daily). Due to low prevalence of this behaviour, this was transformed into a binary variable reflecting any binge drinking in the past six months vs. none. Alcohol-related harms were measured using a 9-item abridged form of the Rutgers Alcohol Problem Index (RAPI) [44]. This measured the frequency (from Never to More than 6 times) over the past 6 months of various harmful consequences of drinking alcohol, such as getting into fights, neglected responsibilities, caused shame or embarrassment. Cronbach’s alpha for the current sample was 0.88. This scale was transformed into a binary variable reflected any alcohol-related harms over the past 6 months compared to none.

### Internalising problems

Depressive symptoms were measured using the 'Patient Health Questionnaire, modified for adolescents (PHQ-A)[17]. One item discussed suicidal thoughts was removed from the PHQ-A as this was not approved by ethics committees, thus the scale comprised 8 items measuring the frequency of depressive symptoms. Scores on each item ranged from 0 (Not at all) to 3 (Nearly every day). Scores were summed to create a total score, ranging from 0 to 24. Scores above 10 met established thresholds for probable depression. Anxiety symptoms were measured by the 'Generalised Anxiety Disorder 7-item scale' (GAD-7) [38]. Scores on each item ranged from 0 (not at all) to 3 (nearly every day) and summed scores ranged from 0 to 21. Scores above 10 met established thresholds for probably anxiety.

### Externalising problems

Externalising problems were measured using the conduct problems and hyperactivity subscales of the Strengths and Difficulties Questionnaire (SDQ) [12]. Each subscale comprises of 5 items, which are scored from 0 (not true) to 2 (certainly true). Each subscale is summed for a total score between 0–10. A summed score of 5–10 in the conduct problem subscale is considered abnormal and a summed score of 7–10 is considered abnormal in the hyperactivity subscale, based on established thresholds [12]. These subscales were confirmed with a five-factor structure that demonstrated satisfactory reliability and validity, predicting an increased likelihood of psychopathology (Goodman, 2001). These same measures were used in a previous Australian trial of Preventure [40].

### Statistical analyses

Quantitative RE-AIM measures were analysed using descriptive statistics. Qualitative data included open-text responses in both the student and teacher surveys. Illustrative quotes were extracted and included in the current manuscript. All open-ended responses are available in the supplement. Student-level effectiveness analyses were conducted in R version 4.3.2. Baseline differences and attrition between intervention and control groups on demographic factors and alcohol use, internalising, and externalising problems were examined using chi-squared analyses for dichotomous variables and Mann–Whitney U-tests for continuous variables, as these variables were non-normally distributed, with a significance threshold for differences set at *p* < 0.05. Substantial attrition occurred over the trial period and was associated with measured variables (see Results section). Data was assumed Missing at Random, and multiple imputation was used to minimise the impact of attrition bias (see Supplement). Mixed-effects regression analyses examined the intervention by time interaction effects, due to the multi-level nature of the data (students nested within schools). Linear models were used for continuous outcomes (internalising and externalising symptoms) and logistic models for categorical data (alcohol use). All models controlled for age, gender (male, female, non-binary or other), and included a nested random effect of students within schools.

## Results

**Fig. 1:**
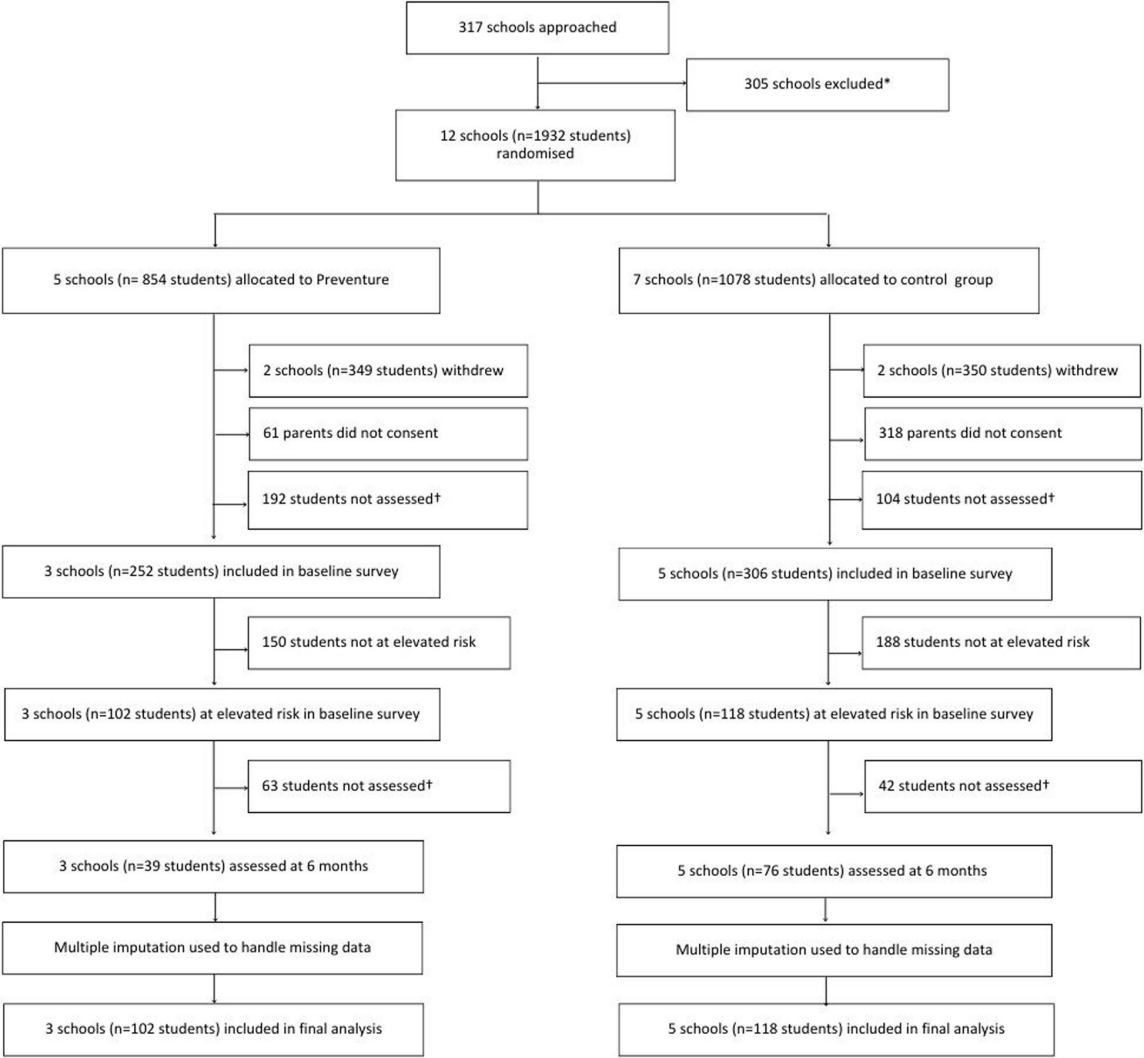
Legend: * Did not respond, declined to participate, or were ineligible given study inclusion criteria. † Did not begin survey

Twelve schools agreed to participate in the study and were randomised, with five schools allocated to Preventure and seven schools allocated to the control group (Fig. 1). However, during the study, four schools withdrew (two intervention, two control; citing the COVID-19 pandemic and associated workload and logistical challenges for schools), leaving a final sample of three intervention schools and five control schools. A total of 558 students completed the baseline survey; of these, 220 students screened as higher than average on one of the personality risk traits and formed our intention to treat sample.

A total of 220 participants had elevated levels of one of the four personality traits on the baseline survey [107 male (48.4%), 101 female (45.7%), 10 non-binary (4.5%) and 2 other identity (0.9%)]. The mean age of the sample at baseline was 13.6 years (SD = 0.7), ranging from 12 – 15 years old. Most participants (*n* = 199; 90.5%) were born in Australia. Baseline characteristics by trial group are provided in Table 1. There were more female students in the intervention group (50.5% female) than the control group (41.5% female), although this difference was not significant (chi square = 0.69, *p* = 0.41; eTable 5). Participants in the intervention group were significantly younger at baseline (median = 13 years) compared to those in the control group (median = 14 years; W = 7964, *p* < 0.01; eTable 5).

There was high attrition at follow-up (48%). While some missing data at follow-up occurred due to participants being absent from school on the day of the survey or declining to take part, missing data was primarily due to COVID-19 impacts and associated teacher burden. School staff reported difficulty allocating class time to complete the surveys as requested by the research team, reporting they needed to prioritise student learning outcomes and support. Instead, in many cases, the research team sent the link to students to complete in their own time, and facilitators reminded the students to complete surveys. Attrition was more likely to occur in the intervention group (39/102 retained, 37.9%) compared to control (76/118 retained, 64.4%); chi square = 15.0, *p* < 0.001; eTable 6), and accordingly, given baseline differences in age, those lost to follow-up were younger (W = 3583.5, *p* = 0.01; eTable 6). None of the primary alcohol, anxiety, or externalising outcomes were associated with attrition. However, those who were lost to follow-up had higher baseline depressive scores than those retained (W = 6804.5, *p* = 0.02; eTable 6).

### Intervention

All 102 students with elevated personality traits in *Preventure* schools were invited to take part in Preventure. An additional 48 students with high scores but below the cut off were also invited to join the groups, in order to ensure adequate group numbers. These students were included in implementation outcomes (that is, provided feedback on the intervention) however, they were not included in the quantitative effectiveness analyses. Across the three intervention schools, six NT groups were run, four IMP groups, four AS groups, and three SS groups. A total of nine students were reported as absent from the Preventure groups, due to being absent on the day. In intervention schools, Preventure was delivered in March 2021, November 2021, September–November 2022. In the greater Sydney area, the most restrictive COVID-19 lockdowns occurred from March to July 2020 and June to October 2021.

Twelve school staff completed the Preventure facilitator training, but one school dropped out post-training, resulting in 8 school staff delivering the Preventure program.

#### RE-AIM implementation outcomes

A total of 61 students (43%) completed the evaluation survey; 18 NT, 18 AS, 12 IMP, 12 SS, and 1 who left blank which personality group they participated in. Open-ended responses were provided by 58 students in total and all responses can be found in eTable 2 in the supplement. Students completing the evaluation were predominantly born in Australia (93%), most reported English to be the main language spoken at home (88%), 60% identified as female, 38% as male, and 2% as non-binary/gender fluid.

Student feedback overall indicated the program was positively received by students who took part in the trial, with 86.9% reporting it was ‘good’ or ‘very good’, and 83.6% reporting the stories in the Preventure program were somewhat relevant or completely relevant to their lives (see eTable 7 and eFigures 1–3 in the supplement). When asked whether they would recommend Preventure to their friends, 67.2% of students said Yes, 27.6% said maybe, and just 5.2% said no.

#### Reach

A total of 150 students were invited to participate in the Preventure program. Of these, 141 completed the intervention (94%).

### Perceived effectiveness

Staff perspectives: As shown in Table 2, all facilitators endorsed Preventure as effective and useful for students, and in general rated the program highly. Responses to open-ended questions regarding any benefits observed by the facilitators included self-identifying and relating with the personality traits, finding commonality with others in the group and initiating friendships with peers in their Preventure group, reduced friendship issues, increased self-awareness and coping skills, understanding how thoughts and actions can impact goal attainment, increased help-seeking, reduced stigma about mental health and development of a shared language to discuss difficulties, increased morale among students, increased engagement with students, and reduced incidents of students presenting with mental health problems. One facilitator noted difficulty reporting on observed benefits to the students, due to the COVID-19 impacts on school attendance.

*“The program is a great idea for young people. It enables them to engage in real life scenarios and develop skills that they may never get a chance to develop. Love the program”.*

**Table 2:** Teacher-perceived effectiveness, adoption, implementation, and maintenance of *Preventure*

EFFECTIVENESS	
*To what extent do you believe the students benefitted from the Preventure program?*	
Not at all	0
Slight benefit	0
Moderate benefit	50
Great benefit	37.5
Extreme benefit	12.5
How effective do you think the Preventure program was for providing coping skills?	
Not at all	0
Slightly	0
Moderately	37.5
Very	50
Extremely	12.5
Do you think students will use the skills they learnt?	
Unsure	0
Not at all	0
Somewhat	50
Quite a lot	25
Definitely	25
How would you rate the overall level of engagement among students?	
Very disengaged	0
Disengaged	0
Somewhat engaged	0
Engaged	62.5
Very engaged	37.5
Overall, how useful do you feel the Preventure program is?	
Not at all useful	0
Not very useful	0
Somewhat useful	0
Useful	62.5
Very useful	37.5
Did you notice any negative effects that could be attributed to the program?	
Yes	25
No	75
ADOPTION	
*Do you work in any other setting with young people?*	
Yes	25
No	75
*Do you think it would be feasible to deliver Preventure in these settings?*	
Unsure	100
No	0
Yes	0
*Barriers to delivering the program*	
None	25
Lack of time	62.5
Lack of support	0
Lack of confidence	0
Parental attitudes	12.5
Student background and attitudes	25
Other (difficulties scheduling due to school timetable/other commitments)	12.5
*Do you think the Preventure program could be implemented in a range of settings?*	
No	0
Yes	100
IMPLEMENTATION*	
*How confident do you feel in your ability to implement the Preventure program?*	
Not at all	0
Slightly	0
Moderately	12.5
Very	75
Extremely	12.5
*How difficult or easy did you find adhering to the manual?*	
Very difficult	0
Difficult	0
Somewhat easy	25
Easy	37.5
Very easy	37.5
*Was there anything that made it challenging to adhere to the manual?*	
Yes	25
No	75
MAINTENANCE	
*Will the Preventure program be implemented in your school in an ongoing basis?*	
No	0
Unsure	75
Yes	25
*Will you continue to be involved in the delivery of Preventure?*	
No	0
Unsure	75
Yes	25
*Do you think the Preventure training workshop was sufficient to enable you to continue delivering Preventure without further training?*	
No	25
Yes	75
*Are there any barriers to the continued implementation of Preventure in your school?*	
No	25
Yes	75
*To what extent do you believe Preventure will become an established program in your school?*	
Very unlikely	0
Unlikely	0
Unsure	62.5
Likely	37.5
Very likely	0

^*^Implementation was measured via i) self-report form completed by the main facilitator, ii) a report made by the co-facilitator, and iii) self-report questions to gage their overall perspectives on how easy or difficult it was to adhere to the intervention. Only responses to iii are presented in this table, further implementation results are presented below and in Table 4

Facilitators reported high student engagement in Preventure: most reported no negative effects could be attributed to the program. One facilitator stated some of the content brought up challenging issues or memories for students (but did not elaborate on these), and while this was not necessarily negative, they thought it important to anticipate this for future sessions. Another facilitator reported that some students discussed experiences far beyond that of other group members, noting that students can be introduced to new risky behaviours through programs such as Preventure that include group sharing.

Student perspectives: When asked an open response question about whether there were any good things about the Preventure Program, 57 students provided a response. Illustrative quotes included *“Showing that negative thinking can impact physical sensations”*; *“I learnt a lot about thinking before doing—consequences, is this going to help me with my goals”*; *“I liked learning about how to cope with these emotions”*; *“We discussed topics that are helpful to us and there was no judgement”*. When asked if there were any bad things about Preventure, of the 50 students who responded, 38 (76%) said “no”. Free-text responses to whether there were any bad things about Preventure included *“It was awkward at times, especially during the silence”*; *“Reading a lot of words”*; and *“Felt uncomfortable with some of the people in my group”*. Other general comments included: *“Have stories about discrimination, pictures of hanging at a friend's house, add more rural images”* and *“Maybe if we could also talk about issues that would really have impacted kids, like COVID, school, and social media”.* All student responses to the open-text fields can be found in eTable 2 in the supplement.

### Teacher-perceived Adoption

Eight school staff delivered the Preventure program as part of the current study (6 teachers, 1 school psychologist, and 1 student support officer/social worker). One facilitator reported less than 2 years' experience working in schools, two facilitators reported 3–5 years' experience working in schools, two reported 6–10 years' experience working in schools, and three reported two more than 10 years' experience working in schools. As shown in Table 3, most staff did not report working in other settings, and for those that did, they were unsure whether it would be feasible to deliver *Preventure* in these settings (the settings were primary schools and sports coaching). The most commonly identified barrier to program delivery was a lack of time.

**Table 3:** Primary outcomes at baseline and follow-up assessments by intervention group

Outcome by assessment time	Control (*n* = 118) N %	Preventure (*n* = 102) N %
*Intention to use alcohol*		
Baseline	70 (59.3)	54 (52.9)
Follow-up	66 (55.9)	56 (54.9)
*Standard drink (past 6 months)*		
Baseline	15 (12.7)	10 (9.8)
Follow-up	15 (12.7)	23 (22.5)
*Any binge drinking (past 6 months)*		
Baseline	4 (3.4)	2 (2)
Follow-up	3 (2.5)	6 (5.9)
*Any alcohol-related harms (past 6 months)*		
Baseline	10 (8.5)	7 (6.9)
Follow-up	8 (6.8)	14 (13.0)
*Depressive symptoms*		
Mean (SD)		
Baseline	8.94 (6.70)	9.74 (6.91)
Follow-up	7.40 (6.36)	8.29 (6.41)
*Anxiety symptoms*		
Mean (SD)		
Baseline	7.16 (6.25)	8.40 (6.85)
Follow-up	6.02 (5.95)	7.42 (6.20)
*Conduct problems*		
Mean (SD)		
Baseline	3.50 (1.85)	3.20 (1.84)
Follow-up	2.88 (1.76)	2.92 (1.79)
*Hyperactivity*		
Mean (SD)		
Baseline	5.60 (2.42)	5.55 (2.36)
Follow-up	5.55 (2.43)	5.23 (2.25)

### Implementation

Facilitators from three schools provided fidelity data after completing a total of 15 Preventure workshops (3 AS, 6 NT, 3 SS, and 3 IMP). As shown in eTable 4, co-facilitator-rated adherence scores were high, indicating that co-facilitators rated the main facilitator as covering program material and engaging students. Constraints to adherence noted by co-facilitators tended to be time constraints, such as having less than the required 90 min to run the Preventure workshop because of students’ other commitments (e.g., sport). Facilitators also rated their adherence to the program highly. For both facilitator self-rated and co-facilitator rated, adherence scores were lower in the impulsivity groups.

The main barrier to program delivery reported by facilitators was time. Facilitators also noted that many of the difficulties they experienced with program delivery were related to aspects of the research trial (such as having to chase parents to obtain consent, time required for the baseline survey), and COVID-19:

*“The way the program was run in the trial will differ from the way we would run it in terms of surveying all students initially, then making it an "opt out" rather than a chasing for permissions from parents. This was too time consuming and unnecessary considering parents are informed and can always choose not to have their child involved in the wellbeing activities of the school during the year. It also meant some of the students we know who really needed it were not allowed to continue out of a parental fear of where the information was going”.*

All staff reported confidence in implementing and adhering to Preventure, although one facilitator found the facilitator guide unhelpful:

“Too much information/too wordy …. To keep engagement of students, I needed to be more dynamic and not focused on the facilitator manual.

Another facilitator noted logistical barriers would be lessened if they just had one facilitator, rather than two as recommended (facilitator and co-facilitator), stating that teachers are used to dealing with larger groups.

All facilitators agreed Preventure could be delivered in a range of settings, and provided suggestions including school camps, youth groups, youth centres and other youth community settings, and youth mental health settings. They noted some potential difficulties with delivering Preventure in non-school settings, such as logistics in screening and obtaining parent/guardian consent, and whether the program would be appropriate for adolescents with more acute/severe mental health issues.

### Teacher anticipated Maintenance

Despite highly positive ratings overall, only two facilitators reported that Preventure would be delivered in their school post-trial, with the remaining six facilitators reporting uncertainty about the ongoing delivery of Preventure in their school. The main barrier suggested for ongoing implementation of Preventure was time. Other potential barriers noted financial barriers (e.g. casual staff to cover teachers for both the administrative and delivery components of the program), and room availability.

*“Our staff who led the program are keen to run it again as we can see the benefit to the students. We would have to make it more timely and user-friendly though, to fit our timetable."*

As shown in Table 2, while most of the facilitators confirmed that the Preventure training workshop was sufficient to enable them to continue delivering Preventure without further training, two facilitators thought the training was not sufficient. One person suggested that further refresher training would be helpful, considering the instability due to COVID-19, and the other suggested breaking the training up over multiple days, and making it more applied, such as completing the screener with students’ in-between sessions, so that they could use real data in the training and be assisted with forming groups as part of the training.

#### Student-level effectiveness outcomes

As described previously, attrition was more likely to occur in the intervention group compared to control, and those lost to follow-up were younger, and had higher depressive symptoms at baseline compared to those retained. Given these differences, multiple imputation was used to minimise the impact of attrition bias (details available in the supplement). Raw frequencies and mean prevalence in outcomes over time are provided in Table 3.

### Alcohol use

At baseline, 30.6% (*n* = 67) had ever drunk alcohol. Of those, most had consumed at least a full standard drink (85.1%, *n* = 57) and just under half (37.3%, *n* = 25) had consumed a standard drink in the past 6 months. The prevalence of binge drinking was low at baseline, with 6 participants (2.7%) endorsing binge drinking in the past 6 months. Most participants indicated it was likely or very likely that they would try alcohol at any time in the future, or had already tried alcohol (56.0%, *n* = 123) and 44% (*n* = 97) thought it unlikely or very unlikely. Most of those who had consumed a full standard drink in the past 6 months endorsed alcohol-related harm over the past 6 months (*n* = 17, 68%).

### Internalising symptoms

At baseline, depressive symptoms on the PHQ-A ranged from 0–24, with a mean of 9.3 (SD = 6.8). Overall, depressive scores decreased by follow-up, with a mean of 7.8 (SD = 6.4). Baseline anxiety scores ranged from 0–21 on the GAD-7, with a mean of 7.7 (SD = 6.6). Similarly, anxiety scores decreased overall by follow-up, with a mean of 6.7 (SD = 6.1).

### Externalising symptoms

At baseline, scores ranged from 0–9 on the conduct problems subscale of the SDQ, with a mean of 3.7 (SD = 1.9) and 0–10 on the hyperactivity scale of the SDQ, with a mean of 5.6 (SD = 2.4). By follow-up, the mean of conduct problems was 2.8 (SD = 1.8) and 5.1 (SD = 2.6) on the hyperactivity scale.

Table 4 shows the results of the mixed effects logistic (alcohol use outcomes) and linear regression models. As shown, there was no significant main effect of the intervention, nor any significant interaction by time effects for any outcomes. However, there was a significant effect of time, with students in both intervention and control schools showing reduced depression scores at follow-up (b = -1.47, SE = 0.73, *p* < 0.05), as well as conduct problems (b = -0.62, SE = 0.24, *p* < 0.05).

**Table 4:** Mixed effects regression examining main effects of the intervention, time, and interaction by time effects

	Main effect intervention b (SE)) p	Main effect time b (SE)) p	Intervention x time interaction b (SE)) p
Intention to use alcohol	-0.22 (0.40) p = 0.59	-0.11 (0.59) p = 0.75	0.35 (0.48) p = 0.47
Standard drink	-0.25 (0.83), p = 0.77	b = 0.10 (0.59) p = 0.86	b = 1.23 (0.87) p = 0.16
Binge drinking	b = -1.95 (2.96) p = 0.51	b = -0.65 (1.47) p = 0.66	b = 3.45 (2.40) p = 0.16
Alcohol-related harm	b = -0.25 (0.75) p = 0.74	b = -0.38 (0.69) p = 0.58	b = 1.30 (1.01) p = 0.20
Depressive symptoms	b = 0.61 (0.93) p = 0.51	**b = -1.47 (0.73) p < 0.05**	b = 0.10 (1.17) p = 0.93
Anxiety symptoms	b = 1.16 (0.84) p = 0.17	b =—1.05 (0.72) p = 0.14	b = 0.10 (1.16) p = 0.93
Conduct problems	b = -0.18 (0.31) p = 0.55	**b = -0.62 (0.24) p < 0.05**	b = 0.29 (0.36) p = 0.43
Hyperactivity	b = -0.06 (0.33) p = 0.87	-0.09 (0.26) p = 0.72	b = -0.17 (0.40) p = 0.67

## Discussion

The current study was an implementation RCT of school staff-led delivery of the *Preventure* program. Guided by the RE-AIM framework, we found the program was well-liked by both teachers and students. With respect to reach, we found strong potential of the program, with 94% of invited students completing the intervention. Staff and student perspectives of the perceived effectiveness of the program were also high, with all staff believing the program was of benefit to the students, was effective in providing coping skills, that students would use the skills they learned, and that the program was useful. Barriers to adoption were generally structural factors, such as dedicated time and the COVID-19 pandemic, rather than elements of the program itself. Staff showed good adherence to the intervention and generally rated the program as easy to implement. Despite these positive ratings, the anticipated maintenance of the program in schools was low: only a quarter of the teachers surveyed said *Preventure* would be implemented in their school in an ongoing basis, largely due to time constraints.

Regarding the effectiveness of the intervention on student outcomes, we did not find that Preventure significantly affected any of our primary outcomes. This should be considered in light of substantial attrition and lack of power; thus, caution should be taken when drawing conclusion about effectiveness from the current study. However, across all internalising and externalising outcomes, we did observe high levels at baseline which reduced over time, with depressive symptoms and conduct problems showing significant declines from baseline to follow-up for both intervention and control groups. This effect of time may reflect the changing context with respect to the COVID-19 pandemic, such as less uncertainty about the impact of infection for children as time progressed and the development of COVID-19 vaccines that signalled a potential future easing of stay-at-home restrictions and a return to relative normalcy. While positive, this may have made it harder to find effects of the intervention, especially as the trial was underpowered due to the severe impact of COVID-19 on recruitment and retention of schools in the trial. Finally, COVID-19 necessitated amendments to our original study timelines, resulting in only a 6-month follow-up. This is a very short period to measure preventive effects, particularly for alcohol use outcomes which emerge around a two-year follow-up [40]. Prevention effects for alcohol use may not emerge until key developmental periods, such as when most young people begin drinking alcohol – 16.1 years for Australians in 2022–23 [2]. Moreover, students likely need time to practice and implement the skills learned in their own lives, which may help to explain why effects are observed far in the long-term (e.g. seven years post-intervention) [31] but not in the immediate term. Finally, this trial was conducted during a unique context of the COVID-19 pandemic, characterised by unprecedented disruptions to schools and young people’s lives and high uncertainty and anxiety. It is plausible that during the pandemic, single interventions aren’t sufficient to shift mental health symptoms. Adolescents also had reduced opportunities to socialise with peers in unsupervised environments and less chance to engage in risky drinking, resulting in less room to observe preventive effects for drinking outcomes.

Despite frequent concerns in the literature about the potential for stigma involved in selective prevention, it is important to note that none of the students reported feeling stigmatised about being selected into the groups, but rather made comments such as *“There was no judgement”.* The potential for stigma was taken very seriously by the research team, who worked with schools during training to discuss the importance of how the program was framed to students and other staff within the school. For example, staff were encouraged to schedule the *Preventure* sessions at the same time as running general wellbeing or goal setting workshops for all students, framing invitations to participate in *Preventure* groups as to engage in goal setting activities with students with similar personality traits. The program itself and communication surrounding it does not speak to elevated risk of mental health and substance use problems, but rather that it can help young people manage their personality styles to align with their long-term goals. That no students reported feeling stigmatised or labelled suggests that screening procedures and selective programs are acceptable and feasible within the school context.

Staff feedback highlighted that even though Preventure was well-liked, staff reported they were overwhelmed by the tasks involved in implementing the program in their school. This was likely exacerbated by the COVID-19 pandemic [3, 34]. The timing of the School-led Preventure study, beginning in 2020 during the height of COVID-19 pandemic restrictions meant implementation of the program by staff was particularly challenging. Thus, the question remains somewhat unanswered as to whether *Preventure* can be feasibly delivered by school staff in the post-COVID-19 context. However, even without the additional burden of the COVID-19 pandemic, teachers have increasingly been reporting high stress, burnout and limitations to resources [35], von [42] and that strong organisational support is needed for program implementation within schools [28]. As such, if the *Preventure* program was to be successfully facilitated by staff in the future, it would benefit from broader organisational support, improved working arrangements and additional resources provided to teachers. It should be noted that the research trial itself contributed to the time burden on teachers, and this would be eliminated outside the context of a trial. For example, many staff expressed that the requirement for active parent consent was not the norm for programs run at the school and resulted in substantial extra time commitment from staff to follow-up parent consent, as well as potential inequitable access to the program, given students who might benefit were the ones less likely to receive active parent consent. Ethical approval committees may need to reconsider whether active parental consent is required for programs that have already undergone rigorous evaluation and pay more attention to the inequities and biases that active parental consent may create. Additionally, students would not be required to complete surveys outside the context of a research trial, further minimising the time commitment for teachers to arrange these surveys and follow-up with missing students.

There are several paths forward for school-based prevention of mental health and substance use among adolescents. One promising option is to have dedicated time or roles to focus on student wellbeing, where programs such as *Preventure* are a core part of the role. This is aligned with the Student Support Officer model in New South Wales, Australia, where staff are embedded within schools to focus on student wellbeing but do not have a teaching load [18]. Another potential for schools is the implementation of universal programs, which are delivered to all students in a cohort regardless of pre-existing risk. Because of this, universal programs can be easier to implement and may be more feasible to deliver at scale compared to selective prevention programs. They are also often preferred by schools themselves, aligning with school priorities to address mental health in all students [4]. A previous trial comparing universal, selective, combined universal and selective, and active control for alcohol use demonstrated that universal and selective approaches were equally as effective for preventing alcohol use compared to control [40]. Despite this promise, the literature on universal prevention programs for depression is disappointing, with outcomes ranging from null and iatrogenic effects to, at best, small effect sizes for symptom reduction observed in the short-term, but rarely sustained beyond 12 months [1, 15, 21, 29, 41, 43]. Efforts to improve universal school-based prevention of depression are an area of critical importance [5].

This study has many strengths, including the prospective registration, cluster randomised design, and attention to both effectiveness of *Preventure* on student outcomes as well as feasibility to implement by school staff. However, the results of this study should be interpreted in light of several limitations. Firstly, there was substantial attrition across follow-up, and attrition was more likely to occur in the intervention group, and among those with higher depression scores at baseline. This greater attrition in the intervention group may be a result of the additional time required for staff in the intervention schools to engage in training and deliver the program to students, leaving less time to coordinate the data collection surveys and chase up missing students. The analyses involved multiple imputation to minimise attrition bias, however given the amount of missing data there is potential for attrition to have influenced study results. Secondly, the COVID-19 pandemic significantly impacted the trial. Due to substantial delays in recruitment and school withdrawals due to the pandemic, we did not reach our required sample size and were thus underpowered to observe an effect of the intervention. The final follow-up timepoint (12-months post-baseline) additionally had to be dropped to accommodate delays in trial activities while schools shifted to remote learning at multiple times throughout the trial. This left only a 6-month window to observe effects for alcohol use, internalising and externalising symptoms. Prevention effects take time to emerge, and 6 months is unlikely to be long enough to observe any preventive benefit associated with the intervention. In addition, to ensure adequate numbers for the Preventure groups, some groups included students who scored highly but below established cut-offs on the screening tool. This could have influenced the intervention, particularly around group discussions, potentially altering the dynamic which typically reflects a collegiality and shared experience of coping with a specific personality trait. Alternatively, as one teacher speculated, it is possible that some students may be introduced to risky behaviours through group sharing by other students with more elevated personality scores. While important to acknowledge this potential, previous trials of Preventure have not seen any increase, but rather demonstrated decreases, in risky behaviours [31, 32]. The content of Preventure focuses on learning about the costs of risky behaviours, acknowledging that students may come across these behaviours eventually and that it is safer to work through potential consequences and alternatives in a structured and safe environment so that students are less likely to take these up in future. Another limitation was the use of trial-specific measures for collecting data on student alcohol use, as well as self-report surveys to capture our primary outcomes of alcohol use, internalising and externalising symptoms, and many implementation effectiveness outcomes. These are subject to recall and social desirability bias. In addition, the RE-AIM measures were collected from teachers, who are typically not the decision-makers on program adoption and maintenance. These measures would be better collected from school administrators. Moreover, in the current study these constructs are limited to teacher-reported *perception of potential* adoption and maintenance, whereas within RE-AIM these refer to actual adoption and maintenance over time. Additionally, the researchers chose certain open-ended responses from school staff and students to highlight in this manuscript. While the most illustrative quotes were chosen, a formal thematic analysis was not conducted, and the selection of quotes potentially reflects the authors’ positionalities. All open-ended responses can be found in the supplement. Finally, schools were recruited from the greater Sydney region only and were generally high SES metropolitan areas. Thus, our results should not be considered representative of the population, and schools in more under-resourced areas may face additional challenges with implementing selective alcohol use and mental health prevention programs.

Overall, the findings of this study demonstrate many strengths of the *Preventure* program for schools: it is well-liked by students and teachers and both groups perceived benefit for students. Teachers are confident in delivery and demonstrate good adherence to the intervention. While we did not observe benefits of the intervention for students in alcohol use, internalising or externalising symptoms, the trial was underpowered and could only involve a short-term (6-month follow-up), given the severe impact of the COVID-19 pandemic. A lack of time was a huge barrier reported by teachers, particularly in the context of the COVID-19 pandemic. Greater resourcing and support would need to be provided if teachers are to be involved in program delivery.

## Supplementary Information

Supplementary Material 1.

## Data Availability

The datasets generated and/or analysed during the current study are not publicly available due to conditions of ethical approval.
