# Supplementary material for: A randomised controlled implementation trial of the feasibility and effectiveness of school staff delivery of a selective substance use and mental health program during the COVID-19 pandemic
Source: BMC Public Health. 2025 Jan 28;25:349. doi: 10.1186/s12889-025-21493-1 (PMC11773974; doi:10.1186/s12889-025-21493-1)
Supplement: Supplementary file 1 — Supplementary Material 1. [file 12889_2025_21493_MOESM1_ESM.docx]

Supplement

eTable 1. Planned and actual study timelines, and stay-at-home orders affecting the study area.

|  | 2020 | | | | 2021 | | | | | | | | | | | | | | | | | | | 2022 | |  |
| --- | --- | --- | --- | --- | --- | --- | --- | --- | --- | --- | --- | --- | --- | --- | --- | --- | --- | --- | --- | --- | --- | --- | --- | --- | --- | --- |
|  | Mar-Jul | Oct | Nov | Dec | Jan | | Feb | | Mar | Apr | | May | Jun | | Jul | Aug | | Sep | Oct | | Nov | Dec | | Jun | | Dec |
| Planned |  | Baseline | | | | | | | | Post-intervention | | | | | | | | | 12-month | | | | |  | |  |
| Actual |  |  |  | Baseline | | | | | | | | | | | | | | | | | | | | | |  |
|  |  |  |  |  |  |  | |  | |  |  | | |  |  | |  | Post- intervention | | | | | | | | |
|  |  |  |  |  |  |  | | Preventure | |  |  | | |  | | | | | | Preventure | | |  | | Preventure | |
| COVID-19 lockdown | Study areas in lockdown |  |  |  |  |  | |  | |  |  | | | Study areas in lockdown | | | | | |  | | |  | |  | |
| Other events | Pandemic declared* (18 March) |  |  |  |  | First COVID-19 vaccination in AUS (22 Feb) | |  | |  |  | | | Sydney schools remote learning | | | | | | Schools go back to face-to-face learning | | |  | |  | |
|  | NSW schools remote learning (24 Mar –25 May) |  |  |  |  |  | |  | |  |  | | |  | | | | | |  | | |  | |  | |

* Governor-General declares a human biosecurity emergency period.

RE-AIM questionnaire for teachers

*We would really appreciate your feedback on the implementation of the Preventure program in your school. Please answer the questions below and feel free to add your own comments at the end of the questionnaire.*

**Expertise**

1. Where is your school located? (i.e. suburb/region)

_______________________________________________________

1. What is your profession?
   - Teacher
   - Mental Health Nurse
   - Psychologist
   - School Counsellor
   - Other - Please specify: _______________________________
2. How many years of experience do you have working in schools?
   - 2 or less
   - 3-5
   - 6-10
   - More than 10
3. Do you work with culturally and linguistically diverse students?
   - Not at all
   - Rarely
   - Somewhat
   - Often
   - Exclusively
4. Do you work with Aboriginal and/or Torres Strait Islander students?
   - Not at all
   - Rarely
   - Somewhat
   - Often
   - Exclusively

**Effectiveness**

1. a) To what extent do you believe the students benefitted from the Preventure program?

| 0 | 1 | 2 | 3 | 4 |
| --- | --- | --- | --- | --- |
| Not at all | Slight benefit | Moderate benefit | Great benefit | Extreme benefit |

b) If 1 or higher, please describe the benefits you observed below:

__________________________________________________________________________________

__________________________________________________________________________________

1. How effective do you think the Preventure program was in providing students with coping skills to manage their personality style?

| 0 | 1 | 2 | 3 | 4 |
| --- | --- | --- | --- | --- |
| Not at all | Slightly effective | Moderately effective | Very effective | Extremely effective |

1. Do you think the students will use the skills learnt in the Preventure program in their lives and to manage their personality styles?

| 0 | 1 | 2 | 3 | 4 |
| --- | --- | --- | --- | --- |
| Unsure | Not at all | Somewhat | Quite a lot | Definitely |

1. How would you rate the overall level of engagement among students during the Preventure program?

| 0 | 1 | 2 | 3 | 4 |
| --- | --- | --- | --- | --- |
| Very disengaged | Disengaged | Somewhat engaged | Engaged | Very engaged |

1. Overall, how useful do you feel the Preventure program is?

| 0 | 1 | 2 | 3 | 4 |
| --- | --- | --- | --- | --- |
| Not useful at all | Not very useful | Somewhat useful | Useful | Very useful |

1. Did you notice any negative effects that could be attributed to the program?

No

Yes - Please specify: _____________________________________________________________

_______________________________________________________________________________

1. Did your students receive any other program/coping skills during the time they completed the Preventure program?

- No
- Unsure
- Yes - Please specify: _______________________________________________________

1. Did you notice any benefits within the school, or grade as a whole (i.e. including students that did not receive the intervention) that could reasonably be attributed to the Preventure program?

No

Yes - Please specify: _____________________________________________________________

**Adoption**

1. Do you work in any other settings with young people? e.g. different schools, organisations
   - No
   - Yes - Please specify: _______________________________________________________
2. If yes, do you think it would be feasible to deliver Preventure in these settings?
   - Yes
   - Unsure
   - No – Please specify why you do not think it would be feasible to implement Preventure in these settings: ________________________________________________________________________

___________________________________________________________________________

1. If you have come across any barriers to delivering the program, please tick any that apply from the below list, or add additional barriers:
   - Lack of time
   - Lack of support
   - Lack of confidence
   - Parental attitudes
   - Student background & attitudes
   - Other, please specify: ______________________________________________________
2. How could implementing Preventure have been made easier for you, either by the researchers or your workplace?

_______________________________________________________________________________

_______________________________________________________________________________

**Implementation**

1. How confident do you feel in your ability to implement the Preventure program?

| 0 | 1 | 2 | 3 | 4 |
| --- | --- | --- | --- | --- |
| Not at all | Slightly confident | Moderately confident | Very confident | Extremely confident |

1. How difficult or easy did you find adhering to the manual?

| 0 | 1 | 2 | 3 | 4 |
| --- | --- | --- | --- | --- |
| Very difficult | Difficult | Somewhat easy | Easy | Very easy |

1. Was there anything that made it challenging to adhere to the manual?

- No
- Yes - Please specify: _______________________________________________________

1. Do you think the Preventure program could be implemented as per the program instructions and manual in a range of settings? (i.e. settings other than schools)

- No
- Yes - Please specify which settings: ___________________________________________

___________________________________________________________________________

1. Do you think there are any aspects of the delivery of the program that would not be practical in other settings?

- No
- Yes - Please specify which aspects of the program would not be practical in other settings: ________________________________________________________________

___________________________________________________________________________

1. Are there any other comments you would like to make in relation to the recent implementation of Preventure at your school? Or in general, about the implementation of the Preventure program?

____________________________________________________________________________

____________________________________________________________________________

**Maintenance**

1. Will the Preventure program be implemented in your school on an ongoing basis?
   - Yes
   - Unsure
   - No - Please specify why not _____________________________________________
2. Will you continue to be involved in the delivery of Preventure?
   - Yes
   - Unsure
   - No - Please specify why not ______________________________________________
3. Do you think the Preventure training workshop was sufficient to enable you to continue to deliver Preventure without further training?
   - Yes
   - No - Please specify why not ______________________________________________
4. Are there any barriers to the continued implementation of Preventure in your school?
   - Yes - Please specify: _____________________________________________________
   - No
5. How much administration time was required to successfully implement the Preventure program? (in total) ___________________________
6. Were there any costs involved in implementing the Preventure program?
   - Yes - Please specify: _____________________________________________________
   - No
7. After the research trial has finished (mid 2021), how many times per year do you (or does your school) intend to deliver Preventure? __________________
8. To what extent do you believe that Preventure will become an established program delivered in your school? ________________________________________________________________

Any other comments:

____________________________________________________________________________

____________________________________________________________________________

____________________________________________________________________________

*Thank you for your time*

eTable 2: Full open-ended responses of students to the Preventure program.

| **Were there any good things about Preventure?** | **Were there any bad things about Preventure?** | **Do you have any other comments?** |
| --- | --- | --- |
| Just understanding it more I guess | No | No |
| Good information content | It was uncomfortable sharing in front of a group setting |  |
| Yes, it helped me realise how to control my thoughts so I didn't get so anxious | No | Maybe if we could also talk about issues that would really have impacted kids, like covid, school, and social media |
| It was a safe place to open up about how you're feeling without judgement | It was a bit awkward at first |  |
| It made me feel like I wasn't alone It taught me how to deal with anxiety | No! |  |
| Yes - people | No | No |
| Yes, we got to eat | No | No |
| Helping to deal with anxiety | No | It was great |
| It was comfortable to talk in a small group of people | Having to talk | Nope! |
| Yes - food and good stories | No | Yes, it's amazing |
| Yes, food | No | Nope |
| Yes | No | It was good |
| We had humorous people in our group | No | No |
| Being with my friends | No | No |
| Very easy to understand | No | No |
| Yes, learning about how to deal wit hanxiety | No | My favourite program |
| Yes, it being relatable | No | It was very good |
| Yes, learnt how to cope better | No | No |
| That we talked over the things that make us impulsive | No | No |
| Being able to talk and not feel like I was getting judged | It was awkward at times, especially during the silence |  |
| Yes, it was very well made and easy to understand | No | No |
| Yes, easy to relate to | No | No |
|  |  |  |
| Learning new things | No | No |
| Food and people in the group | No | No |
| I liked learning about how to cope with these emotions | I thought some of the information wasn't relevant to my situation | No |
| No | Yes, it was boring | No |
| There was good food and helpful information |  |  |
| Yes food | Nothing | No |
|  |  |  |
| Yes, realistic situations |  |  |
| It was funny | Reading a lot of words |  |
| Yes, being able to talk to people about things that they also understand | No | No |
| Yes, the relatable scenarios | No | No |
| I learnt that I am a negative thinker and how to deal with situations where I think negatively | Not really |  |
| I liked that the teachers were nice and chill | I don't know | No :) |
| Yeah, talking to some people | No | No |
| Showing that negative thinking can impact physical sensations | No | no |
| I liked many of the messages given and explained in the program |  |  |
| Yeah, it helps you think reasonably | No | No |
| I got to connect with other kids | We needed more food | Maybe try coming up with stories that go a bit more into depth |
| It was fun The food |  | No |
| Taught you positive thinking | No | No |
| We discussed topics that are helpful to us and there was no judgement |  |  |
|  | Helping us focus on our goals |  |
| I liked how we could explain what could help us | No | No |
|  |  |  |
| The food |  | The food was good |
| Open discussion and the confidentiality Respecting others |  |  |
| Yes | No | no |
| Yes, the group conversation about the material | No | It was great to hear all the strips and explanations |
| I learnt a lot about thinking before doing - consequences, is this going to help me with my goals | No | No |
| Getting to understand why you feel these emotions and being able to relate | No | I think it's a great program as it helps others recognise what they, or their peers, are going through |
| I feel like I understand my personality a lot more after the program |  | No |
| I understand some things about myself | no | no |
| Yes, I can now think more rationally about things | No bad things |  |
| It was specified for certain personalities | No | No |
| It was good to reflect | No | No |
| It gave me a better understanding to deal with hard situations | No | No |
| Yes, we missed out on class and got food | No | No |
| Learning about myself | Felt uncomfortable with some of the people in my group | Have stories about discrimination, pictures of hanging at a friend's house, add more rural images |

eTable 3: Staff responses to open-ended questions.

| Please describe the benefits you observed. | Please specify any negative effects that you noticed. | Did you notice any benefits within the school, or grade as a whole? | What barriers are there to this program? | Please specify | How could implementing Preventure have been made easier for you, either by the researchers or your workplace? | Please specify (anything that made it challenging to adhere to the manual?). | Are there any other comments you would like to make? | Please specify why (training workshop was not sufficient to enable you to deliver Preventure without further training?) | Please specify (barriers to the continued implementation of Preventure in your school?) | Do you have any other comments? |
| --- | --- | --- | --- | --- | --- | --- | --- | --- | --- | --- |
| I have not worked directly with those students following intervention, so unsure, but they seemed engaged in the intervention and I would hope most would continue using the skills. |  | Unsure | Time Student background and attitude |  | The initial survey that students completed took much longer than anticipated. This meant that the survey was started in one class, and completed in another class- impacting on more teachers' lessons, as well as a catch up session. Knowing how long the initial survey would take would have helped us plan better. |  | no |  | Finding the time |  |
| Students identified many elements of the program with their own personalities. | Some content brought up challenging issues or memories for students. Whilst not necessarily negative, it should be anticipated in future sessions. | Some students initiated friendships with the peers in their Preventure groups, so there may have been some kind of influence there. | None |  | More time provided by workplace to prepare to conduct sessions | Interruptions of session by workplace demands e.g. students knocking at door. | Thank you for the opportunity to participate. | Futher refresher training, considering the instability of the recent year would be beneficial. | Time Funding for release time |  |
| Increased awareness and skills to deal with issues that may present in life. |  | Yes, general moral was improved and friendship issues decreased. It is difficult to comment here as COVID impacted school attendance for 10wks in term 3. | Time |  | More time at school to organise the program and hopefully include more students. |  | The program is a great idea for young people. It enables them to engage in real life scenarios and develop skills that they may never get a chance to develop. Love the program. |  | Time allocations for teachers. |  |
| Increased self-awareness. Understanding of how thoughts and actions can hav a positive (or negative) impact on individual goals. |  | I noticed an increase in student engagement with me (even from last year's students) because we went through the same thing together and can relate well due to similar personality traits. | Time Student background and atitude |  | The timing of the program needs to be guided by school timetables and (for us) available space. It was also costly to withdraw the number of staff it required. I do not think it required 2 staff per group. As teachers, we are used to dealing with larger groups and so the program could have been delivered to the (necessary) small group by 1 teacher. | Too much information/too wordy to take the group through with out reading as I went. To keep engagement of students, I needed to be more dynamic and not focused on the facilitator manual. I generally stuck to the workbook and it's logical conclusions as I knew where the program was heading as I went. | Our staff who led the program are keen to run it again as we can see the benefit to the students. We would have to make it more timely and user-friendly though, to fit our timetable. |  |  | The way the program was run in the trial will differ from the way we would run it in terms of surveying all students initially, then making it an "opt out" rather than a chasing for permissions from parents. This was too time consuming and unnecessary considering parents are informed and can always choose not to have their child involved in the wellbeing activities of the school during the year. It also meant some of the students we know who really needed it were not allowed to continue out of a parental fear of where the information was going. |
| Self realisation about personality trait and strategies to overcome negative thinking |  | N/A | None |  | N/A |  | N/A |  | Time and cost of casuals | N/A |
| I think it was helpful for students to see who they'd been grouped with when they found out why. It seemed as though several were surprised they had personality in common with other group member who they would generally have nothing to do with. | I think that some of the students in the group spoke about experinces they'd had which were far beyond those of others. While the program itself didn't introduce any new risky behaviours, the content in the group sharing sessions did. This can be the case in many initiatives like this. | No | Other | It can be hard to make this work during school time. Students who test 'in' need to be withdrawn from other classes (so they miss content, or it is obvious they are elsewhere) and replacing teachers can be difficult at this time. | It was really disjointed with covid and all its complications. I think this would be far easier to run without all the extras that being in the study requires. I'd like to see this run every year. Possibly out of school hours. |  | No | Covid restrictions got in the way. Training would have been better as 2 sessions on 2 days rather than on one long day. We could have facilitated the students survey in between and then used real data to help form groups at second session with advice from course facilitators. | Rooming, teacher covers, finding time in busy schedule. |  |
| Help seeking behaviour, reduction in stigma of talking about mental health/shared language. |  | Unsure | Time Parental Attitude |  | The process for gaining parental consent was the most time consuming; even with online links sent, parents/carers require lots of follow up and prompting to complete. Fitting this in amongst usual workload was difficult. Having uninterrupted time to do this would have been helpful. |  | I think the program content is great. |  | Potentially just time allocation for another staff member to be trained and available for all workshops. | I would like Preventure to be embedded in our wellbeing curriculum; this will just require further conversation/approval from exec. |
| Reduced incidents of students presenting with symptoms of anxiety/ negative thinking |  | Improved interactions between peers who participated in the program together | Time |  | Implement program when schools are not struggling to organise cover for classes. |  | I found preventure to be a helpful tool for students who have previously not understood their personality traits/ behaviours. |  |  | N.A |

eTable 4: Facilitator self-rated and cofacilitator-rated adherence scores of the main facilitator.

| **Co-facilitator rated** | | | | |
| --- | --- | --- | --- | --- |
| **Mean Adherence** | **AS** | **NT** | **SS** | **IMP** |
| Session 1 (0-26) | 24.3 | 24.6 | 26.0 | 19.7 |
| Session 2 (0-21) | 20.3 | 19.0 | 20.7 | 17 |
| **Facilitator self-rated** | | | | |
| **Mean Adherence** | **AS** | **NT** | **SS** | **IMP** |
| Session 1 (0-25) | 24.3 | 24.8 | 25.0 | 20.7 |
| Session 2 (0-25) | 24.3 | 24.8 | 25.0 | 21.5 |

eTable 5. Baseline differences between intervention and control groups.

| **Characteristic** | **χ^2^** | **p** |
| --- | --- | --- |
| Sex | 0.69 | 0.41 |
| Intention to try alcohol | 0.06 | 0.8 |
| Standard drink (past 6 months) | 0.75 | 0.39 |
| Any binge drinking (past 6 months) | 0.15 | 0.70 |
| Any alcohol-related harm | 0.03 | 0.86 |
|  | **W** | **p** |
| Age | 6978.5 | **<.01** |
| Depressive symptoms | 5367.5 | 0.41 |
| Anxiety symptoms | 5116 | 0.17 |
| Conduct problems | 6352 | 0.21 |
| Hyperactivity | 5957 | 0.72 |

eTable 6. Attrition across the follow-up period between intervention and control groups.

| **Characteristic** | **χ^2^** | **p** |
| --- | --- | --- |
| Sex | 0.02 | 0.9 |
| Trial Group | 15.02 | **< .001** |
| Intention to try alcohol | 0.20 | 0.65 |
| Standard drink (past 6 months) | 0.26 | 0.61 |
| Any binge drinking (past 6 months) | 0.68 | 0.41 |
| Any alcohol-related harm | 0.02 | 0.89 |
|  | **W** | **p** |
| Age | 3583.5 | **0.01** |
| Depressive symptoms | 6804.5 | **0.02** |
| Anxiety symptoms | 6549.5 | 0.06 |
| Conduct problems | 5987.5 | 0.62 |
| Hyperactivity | 5238 | 0.21 |

Methods: Multiple Imputation

Multiple imputation and analyses on imputed data were conducted in R version 4.3.2 using the Multivariate Imputation by Chained Equations (mice) package. Using the rule of thumb that the number of imputations should be equal to the percent of missing data, 26 imputed datasets were created that imputed all missing data on baseline and follow-up surveys. Covariates, personality (measured with the SURPS), and all variables included in the present analysis were used to specify the imputed variables. Descriptive analyses were run on all imputed variables to inspect for anomalies. Mixed-effects regression analyses were run on each imputed dataset and pooled using the mice package.

**Student feedback**

eFigure 1. Students rate the Preventure Program overall

eFigure 2: Students report how relevant the Preventure Program stories are to their own life experience.

eFigure 3: Students report how likely they are to use the skills taught in Preventure

eFigure 4: Students report how much they think the skills taught in Preventure will help them cope with their personality trait.

eFigure 5: Students report how helpful they found the information.

eTable 7. Student evaluations of Preventure.

| *How would you rate the Preventure Program overall?* N=61 | % |
| --- | --- |
| Very Good | 32.8 |
| Good | 54.1 |
| Average | 11.5 |
| Poor | 1.6 |
| Very Poor | 0 |
| *How much did you like the stories in Preventure?* N=60 |  |
| Liked a lot | 33.3 |
| Liked a little | 43.3 |
| Neither liked nor disliked | 21.7 |
| Disliked a little | 1.7 |
| Disliked a lot | 0 |
| *How relevant were the stories to experiences in your own life?* N=61 |  |
| Completely relevant | 13.1 |
| Somewhat relevant | 70.5 |
| Unsure | 8.2 |
| Somewhat irrelevant | 6.6 |
| Completely irrelevant | 1.6 |
| *How relevant were the pictures to your own life?* N=61 |  |
| Completely relevant | 9.8 |
| Somewhat relevant | 36.1 |
| Unsure | 42.6 |
| Somewhat irrelevant | 6.6 |
| Completely irrelevant | 4.9 |
| *How helpful was the information on the personality trait to you?* N=61 |  |
| Extremely helpful | 37.7 |
| Somewhat helpful | 50.8 |
| Neither helpful nor unhelpful | 8.2 |
| Somewhat unhelpful | 3.3 |
| Extremely unhelpful | 0 |
| *How easy was it to understand the information in the program?* N=61 |  |
| Very easy | 70.5 |
| Somewhat easy | 19.7 |
| A bit difficult | 9.8 |
| Somewhat difficult | 0 |
| Very Difficult | 0 |
| *How would you describe the feeling in the group?* N=59 |  |
| Very positive group feel | 57.6 |
| Somewhat positive group feel | 16.9 |
| Neutral | 22 |
| Somewhat negative group feel | 3.4 |
| Very negative group feel | 0 |
| *How helpful and understanding were the facilitators?* N=60 |  |
| Extremely helpful and understanding | 61.7 |
| Somewhat helpful and understanding | 28.3 |
| A little helpful and understanding | 8.3 |
| Not very helpful and not understanding | 1.7 |
| *Do you think the skills you received in the Preventure program will help you to deal more effectively with your trait in the future?* N=60 | |
| Yes I think they will help a great deal | 50 |
| Yes I think they will help somewhat | 41.7 |
| No I don't think they will help at all | 3.3 |
| I'm not sure whether they will help | 5 |
| *How likely are you to use the skills taught in Preventure in your own life?* N=59 |  |
| Very likely | 20.3 |
| Likely | 49.2 |
| Unsure | 25.4 |
| Unlikely | 3.4 |
| Very unlikely | 1.7 |
| *Would you recommend Preventure to your friends?* N=58 |  |
| Yes | 67.2 |
| No | 5.2 |
| Maybe | 27.6 |
